# A Single *parS* Sequence from the Cluster of Four Sites Closest to *oriC* Is Necessary and Sufficient for Proper Chromosome Segregation in *Pseudomonas aeruginosa*

**DOI:** 10.1371/journal.pone.0120867

**Published:** 2015-03-20

**Authors:** Paulina Jecz, Aneta A. Bartosik, Krzysztof Glabski, Grazyna Jagura-Burdzy

**Affiliations:** Institute of Biochemistry and Biophysics, Department of Microbial Biochemistry, Polish Academy of Sciences, Warsaw, Poland; Loyola University Medical Center, UNITED STATES

## Abstract

Among the mechanisms that control chromosome segregation in bacteria are highly-conserved partitioning systems comprising three components: ParA protein (a deviant Walker-type ATPase), ParB protein (a DNA-binding element) and multiple cis-acting palindromic centromere-like sequences, designated *parS*. Ten putative *parS* sites have been identified in the *P*. *aeruginosa* PAO1 genome, four localized in close proximity of *oriC* and six, diverged by more than one nucleotide from a perfect palindromic sequence, dispersed along the chromosome. Here, we constructed and analyzed *P*. *aeruginosa* mutants deprived of each single *parS* sequence and their different combinations. The analysis included evaluation of a set of phenotypic features, chromosome segregation, and ParB localization in the cells. It was found that ParB binds specifically to all ten *parS* sites, although with different affinities. The *P*. *aeruginosa parS* mutant with all ten *parS* sites modified (*parS*_null_) is viable however it demonstrates the phenotype characteristic for *parA*_null_ or *parB*_null_ mutants: slightly slower growth rate, high frequency of anucleate cells, and defects in motility. The genomic position and sequence of *parS* determine its role in *P*. *aeruginosa* biology. It transpired that any one of the four *parS* sites proximal to *oriC* (*parS1* to *parS4*), which are bound by ParB with the highest affinity, is necessary and sufficient for the *parABS* role in chromosome partitioning. When all these four sites are mutated simultaneously, the strain shows the *parS*_null_ phenotype, which indicates that none of the remaining six *parS* sites can substitute for these four *oriC*-proximal sites in this function. A single ectopic *parS2* (inserted opposite *oriC* in the *parS*_null_ mutant) facilitates ParB organization into regularly spaced condensed foci and reverses some of the mutant phenotypes but is not sufficient for accurate chromosome segregation.

## Introduction

Accurate DNA segregation to progeny cells is fundamental to the survival of organisms and continuity of life. In Prokaryotes, pioneering studies on the segregation of low-copy-number plasmids have revealed the existence of partitioning systems (*par*) ensuring active distribution of DNA molecules to daughter cells and thus their stable inheritance in bacterial populations [1–3]. The great majority of plasmidic *par* systems comprise three components: an NTPase (component A) that forms a dynamic scaffold for plasmid movement, specific DNA-binding protein (component B), and a *cis*-acting centromere-like sequence(s) recognized and bound by B-component, all together forming a ‘minimalist’ DNA segregation machine [4].

Bacterial genomics has revealed the presence of an operon encoding homologs of type IA plasmidic Par proteins (usually designated ParA and ParB) in close proximity of the origin of replication, *oriC* [5–7], in the vast majority of bacteria with the exception of two families of γ-proteobacteria, *Enterobacteriaceae* (e.g., *E*. *coli*) and *Pasteurellaceae* (e.g., *Haemophilus influenzae*) and one family of Mollicutes, *Mycoplasmataceae* (e.g. *Mycoplasma sp*). The highly-conserved multiple copies of *parS*, the *cis*-acting centromere-like sequence, are mainly localized in the *ori* domain comprising 20% of the genome around *oriC* [7], although in some species, e.g., *Bacillus subtilis* and *Pseudomonas aeruginosa*, additional *parS* sequences are dispersed outside the *ori* domain [8, 9]. The hydrolytic activity of ParA, P-loop ATPase with a deviant Walker A motif [10], provides energy and orchestrates the movement of the nucleoprotein complex of ParB bound to its cognate *parS* site(s) [2, 11–14]. The chromosomal partitioning systems participate in the chromosome segregation by orienting the *ori* domain spatially [15–17], directing the newly replicated origins to the cell poles [18–29], compacting the chromosome by creating a platform for SMC loading [30–32], and holding the *ori* domains at the poles until completion of cell division [12, 29, 33–35].

Numerous studies on various bacterial species (with singular or multipartite genomes, with a simple or complex cell cycle) have revealed on one hand the highly conserved nature of the partitioning components, and on the other the participation of *parABS* systems not only in chromosome segregation but also in other vital cell processes in a species-specific manner [36]. The *parABS* systems may be involved in the regulation of replication [15, 27, 37–41], initiation of sporulation [42, 43], septation and DNA translocation [16, 21, 23, 30, 44–46] as well as growth control and cytokinesis [12, 34, 35, 47–53] or motility [54, 55]. Transcriptomic analyses of *par* mutants have demonstrated the role of Par proteins as global transcriptional regulators in *P*. *aeruginosa* [56] and *Vibrio cholerae* [57].

The interactions of ParA and ParB homologues with one another and with other proteins have been studied thoroughly [9, 12, 13, 16, 29–35, 37–39, 49–55, 58, 59]. The interactions of chromosomal ParBs with the centromere-like sequences have been also analyzed, demonstrating their ability to specifically bind *parS*, spread on DNA, form nucleoprotein complexes and transcriptionally silence genes adjacent to *parS* [8–9, 57, 60–62].

Less is known about why there are multiple *parS* sites on the chromosome and the roles they play. The binding site for chromosomal ParB, first identified for Spo0J (ParB) in *B*. *subtilis* [8, 63] as the 16-nucleotide sequence tGTTtCAcGTGAAAAa/g, seems to be highly conserved in the primary chromosomes throughout the bacterial kingdom [7]. The secondary chromosomes of multipartite bacterial genomes possess their own *parABS* systems [7] demonstrating intra- as well as inter-species structural and functional diversity [25, 64, 65]. Whereas the postulated role of ParB interactions with *parS* sequences in the *ori* domain is to form nucleoprotein complexes that facilitate origin separation and their directional movements [25, 27, 33, 44, 45], the significance of ParB binding to the *parS* sites outside the *ori* domain has not been fully evaluated [8, 9, 62].

Our studies on bacterial chromosome segregation have been conducted on the clinically important opportunistic pathogen *P*. *aeruginosa* representing bacteria with a simple cell cycle. It was shown that *P*. *aeruginosa* PAO1161 *parA* and *parB* mutants were non-lethal but demonstrated wide range of pleiotropic defects, such as slower growth rate, higher frequency of anucleate cells (more than 400-fold), impaired motility (swimming and swarming) and abnormal colony morphology [54, 55]. Similar phenotypes were observed in populations of cells overproducing one of the partitioning proteins, ParA or ParB, demonstrating importance of their proper stoichiometry [54, 55]. Likewise plasmidic members of the family [66], ParB of *P*. *aeruginosa* can polymerize and spread along DNA after binding to the centromere-like sequence, causing transcriptional silencing of neighboring genes in a test plasmid [9]. In *P*. *aeruginosa* cells ParB forms large, compact nucleoprotein complexes co-localizing with ori domains, visualized by use of immunofluorescence as 1 to 4 foci, depending on the stage of growth [55]. In *parA*_null_ mutant ParB foci are much weaker and irregularly distributed whereas in various *parB* point mutants, defective in dimerization/ polymerization and interactions with ParA, multiple small foci are dispersed [54, 55, 67, 68]. A transcriptomic analysis of *P*. *aeruginosa par* mutants [56] has revealed changes in the expression of multiple operons indicating an important role of Par proteins (especially ParB) in coordinating different cell processes either directly through interactions with DNA or indirectly through interactions with putative partner proteins (Glabski K., unpublished). Molecular analysis of ParB derivatives has led to the identification of a dimerization domain [9, 55, 67], domains interacting with *parS* [55, 68], and a polymerization domain responsible for spreading around *parS* sequence to form the nucleoprotein complex [68]. Also for ParA of *P*. *aeruginosa* a dimerization domain and a domain of interactions with its partner ParB have been mapped in the central part of the protein [58].

**Fig 1:**
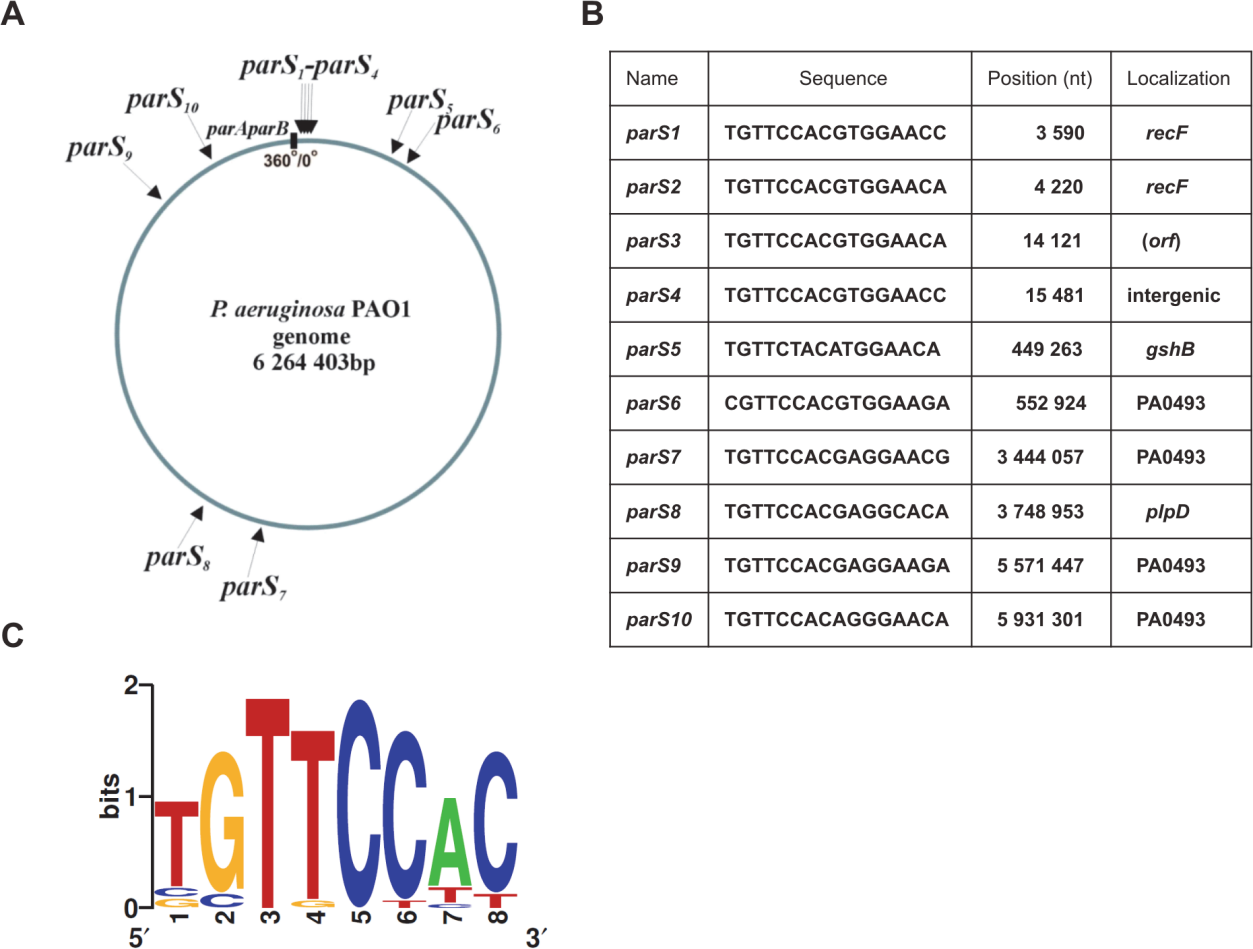
The *parS* sites and their localization in the *Pseudomonas aeruginosa* genome. **(A)** Circular map of the *P*. *aeruginosa* genome with locations of putative ParB binding sequences [9]. Position of the *parAparB* operon is shown as black rectangle, grey arrow marks *oriC*, black arrows indicate predicted *parS* sites. **(B)** Nucleotide sequences, genomic coordinates and gene locations of the *parS* sites. The sequences are presented in a clockwise configuration. The coordinates are given according to the genomic sequence of the PAO1-UW strain [69]. **(C)** Sequence logo for all twenty 8-bp half-sites in the *P*. *aeruginosa* PAO1-UW genome **Error! Bookmark not defined.**). Nucleotides at positions 2 and 5 are invariant in all half-sites.

The *parAB* genes are localized ∼ 7 kb counterclockwise from *oriC* (Fig. 1A) in the reference PAO1 genome [69] and ten putative *parS* sites (numbered clockwise starting from *oriC*) are distributed along the chromosome with eight of them residing in the *ori* domain [9]. Among the *parS* sites, two designated *parS2* and *parS3* are perfect palindromes **TGTTCCAC/GTGGAACA**, *parS1* and *parS4* have one mismatch **TGTTCCAC/GTGGAAC** **C,** and the remaining six have two different mismatches (Table 1). The *parAB* operon and a single perfect palindromic *parS2* sequence have been shown to stabilize otherwise unstable plasmid [9]. *In vitro* tests have demonstrated specific ParB binding to ds oligonucleotides corresponding to *parS1*, *parS2* or *parS7*, with the highest affinity of ParB towards the perfectly palindromic *parS2* oligonucleotide [9]. It has been hypothesized that the varied ParB affinity and localization in/outside the ori domain could be related to the different roles of individual *parS* sequences in *P*. *aeruginosa* biology, e.g., chromosome segregation versus regulation of gene expression.

**Table 1:** Nucleotide substitutions introduced into *parS* sequences.

Name	Wild-type sequence	Mutated sequence	Restriction enzyme
***parS1***	TGTTCCACGTGGAAC***C***	TaTTtCAtGTaGAgC***C***	Eco72I (−)
***parS2***	TGTTCCACGTGGAACA	TGTTtCAtGTaGAgCA	Eco72I (−)
***parS3***	TGTTCCACGTGGAACA	cGTgCCcCGaGGgACg	Eco72I (−)
***parS4***	TGTTCCACGTGGAAC***C***	DELETION	NA
***parS5***	TGTTC***T***AC***A***TGGAACA	TaTTg***T***At***A***TGGAgCA	SacII (−)
***parS6***	***C***GTTCCACGTGGAA***G***A	***C***GTcCCtCGcGGcA***G***g	Eco72I (−)
***parS7***	TGTTCCACG***A***GGAAC***G***	TaTTtCAtG***A***aGAgC***G***	BshTI (−)
***parS8***	TGTTCCACG***A***GG***C***ACA	TaTTtCAtG***A***aG***C***gCA	NruI (+)
***parS9***	TGTTCCACG***A***GGAA***G***A	TaTTtCAtG***A***aGAg***G***A	BglII (+)
***parS10***	TGTTCCAC***AG***GGAACA	TaTTtCAt***AG***aGAgCA	BshTI (+)

Deviation from the perfect palindrome (*parS2/parS3*) are indicated in bold italics, and introduced mutations are in lower case. The mutations destroy (−) or create (+) a restriction site that was used to distinguish between the wt and mutated sequence. NA—not applicable.

Here, we constructed and characterized a set of *P*. *aeruginosa* PAO1161 *parS* mutants with each single *parS* altered and their various combinations including a *parS*_null_ mutant (all ten *parS* sites modified) to shed light on their roles in the cell cycle. *P*. *aeruginosa parS*_null_ mutant is viable although impaired in growth, chromosome segregation and motility. Analysis of mutant strains demonstrated that a single, high affinity ParB binding site in the proximity of *oriC* is necessary and sufficient for accurate chromosome segregation.

## Materials and Methods

### Bacterial strains and growth conditions

The *E*. *coli* and *P*. *aeruginosa* bacterial strains used and constructed in this study, are listed in S1 Table. Bacteria were grown at 30°C or 37°C in L-broth or L-agar (L-broth with 1.5% agar [w/v]). If needed, the media were supplemented with antibiotics: for *E*. *coli* strains benzyl penicillin (Pn) at final concentration 150 μg ml^-1^ in liquid medium and 300 μg ml^-1^ in agar plates, 30 μg ml^-1^ streptomycin (Sm), 50 μg ml^-1^ kanamycin (Km) or 10 μg ml^-1^chloramphenicol were applied; for *P*. *aeruginosa* strains 100 μg ml^-1^ chloramphenicol (Cm), 300 μg ml^-1^ carbenicillin (Cb) and 300 μg ml^-1^ rifampicin (Rif) were used. The L-agar used for blue/white screening contained 0.1 mM IPTG (isopropyl-β-D-thiogalactopyranoside) and 40 μg ml^-1^ X-gal (5-bromo-4-chloro-3-indolyl-β-D-galactopyranoside). Bacterial growth was monitored by measurements of optical density at 600 nm (OD_600_) and c.f.u. ml^-1^ after plating on L-agar.

### Plasmids, oligonucleotides and DNA manipulations

The plasmids used and constructed during this study are listed in S2 Table, applied oligonucleotides are presented in S3 Table. Plasmid DNA was isolated by alkaline method and standard genetic procedures were used as recommended [70]. Chromosomal DNA templates for PCRs were prepared from 1 ml overnight cultures. Pelleted cells were washed with sterile water, re-suspended in 100 μl of sterile ddH_2_0 and boiled. Standard PCR [71] was performed using 5 μl of boiled cell suspension and 5 pmoles of each primer. Fidelity of the amplified DNA sequence was verified by DNA sequencing in the internal sequencing facility (DNA Sequencing and Oligonucleotides Synthesis Laboratory, IBB PAS, Warsaw, Poland).

### PCR site-directed mutagenesis

The QuickChange site-directed mutagenesis method was applied (Stratagene) with the pAKE600 plasmid [72] derivatives containing *parS* sequences as the templates. Only a few nucleotide substitutions were introduced in the primers for the PCR site-directed mutagenesis of intragenic *parS* sites not to modify the amino acid sequences of the orfs. To facilitate the screening of clones with mutagenized plasmid DNA, an appropriate restriction site was disabled or introduced as indicated in Table 1. The PCR-amplified plasmid DNA was treated with DpnI to remove the template DNA and then used for transformation of *E*. *coli* DH5α strain. After the initial screening, the presence of mutations was verified by DNA sequencing of PCR-amplified fragments.

### Bacterial transformation

Competent *E*. *coli* cells were prepared by the standard CaCl_2_ method [70]. Competent *P*. *aeruginosa* cells were prepared as described previously [73].

### Introduction of mutated *parS* alleles into *P*. *aeruginosa* PAO1161 chromosome by homologous recombination

The competent *E*. *coli* S17–1 cells (S1 Table) were transformed with pAKE600 suicide vector derivatives [72] to construct donor strains for conjugation. Bacterial conjugation was done on L-agar by mixing 100 μl of overnight cultures of *E*. *coli* S-17 (pAKE600 derivatives) donors and *P*. *aeruginosa* PAO1161 Rif^R^ recipient strain and incubation for 24 h at 37°C. The bacterial mixtures were washed off the plates with 2 ml of L-broth, and diluted cell suspensions were plated on L-agar with rifampicin and carbenicillin to select for transconjugants/ integrants.

The integrants of PAO1161 Rif^R^ (pAKE600 derivatives) were treated as described previously [54]. The allele integration and then allele exchange was verified by PCR using chromosomal DNA as a template and the adequate pairs of primers. The PCR-amplified fragments with putative mutated *parS* sequences were digested with appropriate restriction enzymes, and also sequenced to confirm the presence of the mutations.

### Purification of His_6_-tagged ParB protein

Exponentially growing *E*. *coli* BL21(DE3) strain with pKLB28 (pET28 derivative, encoding His_6_-ParB) was induced with 0.5 mM IPTG at a cell density of 10^8^ cells ml^-1^ and grown with shaking at 37°C for additional 2 h. The cells were harvested by centrifugation and sonicated in 50 mM phosphate buffer pH 8.0 containing 300 mM NaCl. Overproduced His_6_-tagged ParB protein was purified on Ni-agarose columns (Protino Ni-TED 1000, Macherey-Nagel) with an imidazole gradient in the same buffer. The quality of the purified protein was verified by SDS-PAGE using a Pharmacia PHAST gel system.

### DNA-binding affinity assay

The electrophoretic mobility shift assay (EMSA) was performed according to Ringgaard *et al*. [74]. 6 pmoles of the double-stranded oligonucleotide labeled with the fluorescent dye (Cy3 or Cy5) were incubated with increasing quantities of His_6_-ParB protein in the presence of 18 pmoles of non-specific ds oligonucleotides as a competitor DNA in binding buffer (10 mM Tris-HCl pH 7.5, 0.5 mM dithiothreitol, 50 mM KCl, 1 mM MgCl_2_) [75] in a total volume of 20 μl. In dissociation experiments the constant amount of 240 pmoles of His_6_-ParB was added to 6 pmoles of fluorescently labeled ds oligonucleotides and increasing amounts of unlabeled ds *parS2* oligonucleotide (18, 60, 90, 120, 180 pmoles, respectively). After 15 min incubation at 37°C the samples were separated on 5% polyacrylamide gels in 0.5 x Tris-borate-EDTA buffer (TBE) [70]. The DNA was visualized using FluorChemQ MultiImageIII ChemiImager and the images were captured using Alpha View software (Alpha Innotech).

### ParB silencing test

*E*. *coli* DH5α strain was transformed with pGB2 [76] and its derivatives (with wt or mutated *parS* sequences inserted) selecting for Sm^R^ clones. The transformants cells were made competent and then transformed with an estimated 1 μg of either the pGBT30 (*lacI* ^*q*^ *tacp*) expression vector [77] or its derivative pKLB2 (*lacI* ^*q*^ *tacp-parB*). 100 μl of undiluted and serially diluted transformation mixtures were plated in repetitions on different selection plates. The selection was either for an incoming plasmid only (L-agar with Pn), for both resident and incoming plasmids (L-agar with Pn and Sm) or for both resident and incoming plasmids on plates supplemented with 0.5 mM IPTG to induce ParB production. The ratio of number of colonies on dual selection plates with IPTG versus the number of colonies on L-agar with Pn reflected the strength of ParB-*parS* binding and spreading on DNA (silencing ability).

### Motility assay

For motility assays, *P*. *aeruginosa* PAO1161 derivatives strains were taken from a deep-frozen stock, spread on L-agar plates and grown overnight at 37°C. Then bacteria from single colonies were used to inoculate test plates with sterile toothpicks and such plates were incubated for 24 h at 37°C. For the swimming assay, tryptone plates (1% tryptone, 0.5% NaCl, 0.3% agar) were used; for the swarming test, plates containing 0.5% Bacto agar and supplemented with 5 g l^-1^ of dextrose and 8 g l^-1^ of nutrient broth were inoculated [78]. All sets of plates were standardized by using the same volume of medium. Independent assays were repeated at least three times each strain with wt PAO1161, PAO1161 *parA*_null_ and PAO1161 *parB*_null_ mutants as the control strains.

### Colony morphology

Colonies of *P*. *aeruginosa* strains were observed after 24 h incubation on L-agar plates at 37°C using stereomicroscope Nikon SMZ1500, and images were captured with NIS-Elements 2.10 software.

### Preparation of anti-ParB antiserum

For immunofluorescence microscopy, rabbit anti-ParB antibodies [9] were affinity purified as described previously [79]. Affi-Gel 10 (Bio-Rad) was used as the support for the purified ParB in 20 μl columns made of protein gel loading tips.

### Fluorescence microscopy (DAPI staining and immunofluorescence)

Wild-type *P*. *aeruginosa* PAO1161, *parA*_null_, *parB*_null_, and *parS* mutants were grown in L-broth. At an OD_600_ of 0.4 the cells were collected and used to prepare microscopic slides. Fixing and permeabilization of cells and subsequent 4,6-diamidino-2-phenylindole (DAPI) staining were carried out as described previously [55]. A coverslip was placed on a slide with a 1:4 (v/v) solution of DAPI (1 μg ml^-1^) and Vectashield (mounting medium, Vector Laboratories). Cells were studied with Carl Zeiss Axio Imager.M2 utilizing lens EC Plan-Neofluar 100x/ 1.30 Oil ph 3 M27 and camera AxioCamMR5. The pictures were captured and analyzed with the AxioVision Rel.4.8.2 program (Carl Zeiss). Affinity-purified anti-ParB antibodies (40 μl) were used as the primary antibodies (1:100 dilution in 2% [w/v] bovine serum albumin–PBS), followed by 40 μl of anti-rabbit immunoglobulin G (IgG) conjugated to fluorescein isothiocyanate (FITC) (6.9 μg ml^-1^ in 2% [wt/vol] bovine serum albumin–PBS) (Sigma). The images were analyzed as described above.

## Results

### Cloning of mutated *parS* alleles

Bioinformatic analysis of PAO1 genome [69] has predicted that *parS4* is located in an intergenic region (Fig. 1B) whereas nine other *parS*s are most likely situated within coding sequences. To mutagenize PAO1161, the laboratory strain originated from PAO1, we decided to use deletion to disable *parS4* while for the other sites as many point mutations as possible were introduced without altering the coding sequence. For *parS4* deletion, two fragments of approximately 250 nt each corresponding to the *parS4* flanking sequences were linked in the multi-copy narrow-host-range plasmid pAKE600 [72] to obtain pPJB14 (S2 Table). DNA fragments of approximately 500 nt encompassing each of the other nine *parS* sites were PCR-amplified on PAO1161 DNA using appropriate pairs of primers (S3 Table), cloned into pAKE600 and obtained plasmids (S2 Table) were subjected to PCR-based site-directed mutagenesis. Each pair of mutagenic primers introduced several nucleotide substitutions into a given *parS* and simultaneously destroyed or created a new restriction site to facilitate screening (Table 1). The introduced changes in the *parS* inserts were confirmed by DNA sequencing.

### *In vivo* ParB binding to the modified *parS* sequences

Before introducing the modified *parS* alleles into *P*. *aeruginosa* genome, we verified whether the introduced substitutions affected the ParB binding affinity towards those sites.

**Fig 2:**
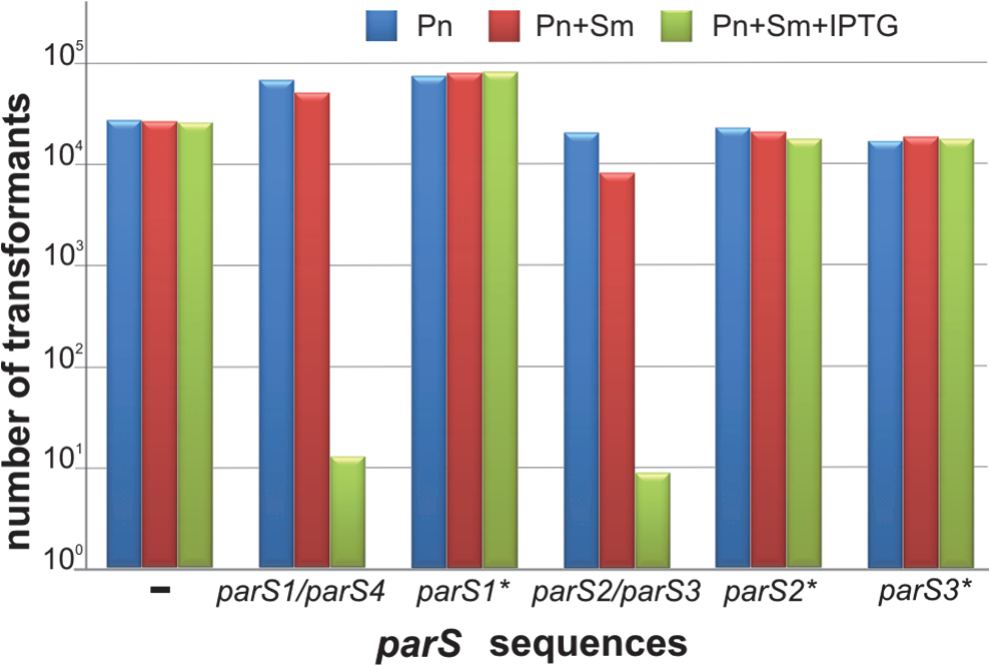
ParB silencing test for wild type and mutated versions of *parS*. *E*. *coli* DH5α transformants carrying pGB2 derivatives with individual wt *parS* sequences or their mutated versions were transformed with pKLB2 (*tacp-parB*_*P*.*a*._). Three independent transformation experiments were conducted and representative results are demonstrated. Undiluted transformation mixtures and their 10- and 100-fold dilutions were plated on three types of selection plates. Numbers of colonies growing on L-agar plates with Pn (blue bar), Pn and Sm (red bar), and double selection plates with 0.5 mM IPTG (green bar) are shown for the undiluted samples.

Upon binding to *parS*, *P*. *aeruginosa* ParB can (as can also plasmidic ParB representatives of group IA) spread along the DNA, thereby silencing the adjacent promoters [9, 66, 68, 80]. It has been demonstrated [9] that when *parS* is cloned upstream of the *repA* gene in the test plasmid pGB2 [76] and ParB excess is supplied from a compatible vector, the plasmid is lost when not selected for. Thus, native *parSs* and their mutated versions were cloned into pGB2 and obtained plasmids were introduced to *E*. *coli* DH5α strain. The Sm^R^ transformants of DH5α carrying the pGB2 derivatives were then transformed with expression vectors: pGBT30 (*lacI* ^*q*^ *tacp*) [77] or its derivative pKLB2 (*lacI* ^*q*^ *tacp-parB*) [9]. Double transformants were selected either for the incoming plasmid (plates with Pn), for both the incoming and resident plasmids (plates with Sm and Pn), or for both plasmids under conditions of ParB overproduction (plates with Sm, Pn and 0.5 mM IPTG). When DH5α (pGB2) with no *parS* cloned was transformed with either pGBT30 or pKLB2, the numbers of colonies were similar regardless of the type of selection plates applied. No incompatibility was observed between pGB2 derivatives containing diverse *parS* sequences and the empty expression vector pGBT30 either (data not shown). However, when DH5α (pGB2-*parS2/par3*) or DH5α (pGB2-*parS1/parS4*) strains (plasmids pABB812 and pABB822, respectively, S2 Table) were transformed with pKLB2, the number of transformants selected on the double selection plates with IPTG was approximately 10^4^-fold lower than the number of transformants growing on plates with Pn only (Fig. 2). This confirmed the previously described [9] inability of pGB2 carrying a perfect palindrome (*parS2/parS3*) or a palindrome with one mismatch (*parS1/parS4*) to replicate in the presence of ParB excess. When DH5α (pGB2-parS1*), DH5α (pGB2-parS2*) or DH5α (pGB2-parS3*) strains (with plasmids pPJB27, pPJB28 or pPJB29, respectively, S2 Table) were transformed with pKLB2 the number of transformants growing under double selection in the presence or absence of ParB excess was the same (Fig. 2). This indicated that those three mutated versions of *parS* sites were not bound by ParB or were bound with too low affinity to block ParB binding/spreading, hence no plasmid loss in the “silencing test” was observed.

Similar transformation experiments (“silencing tests”) were carried out for pGB2 derivatives with native *parS5* to *parS10* insertions (plasmids pPJB22 to pJB26, S2 Table). The number of colonies of DH5α (pGB2-*parS* derivative) (pLKB2) strains when plated on L agar with Pn, Sm and 0.5 mM IPTG was slightly lower (2- to 5-fold) than number of colonies selected on L-agar with Pn, for the incoming plasmid only (data not shown). Such weak “silencing” effect of ParB binding to the wild-type *parS* sites containing two mismatches (*parS5* to *parS10*) indicated that *in vivo* assay would not be sufficiently sensitive to analyze putative differences in ParB affinity between this group of wt *parS*s and their mutated versions.

### *In vitro* ParB binding to wild-type and modified *parS* sequences and the hierarchy of wt *parS* sites

To analyze the effect of the mutations introduced in *parS* sequences on ParB binding, an *in vitro* EMSA test with fluorescently labeled ds oligonucleotides and His_6_-tagged ParB was performed.

Pairs of differently labeled ds oligonucleotides representing each wt *parS* and its mutated version were used in the EMSA. An unrelated ds oligonucleotide was used as a control to test for unspecific DNA binding by ParB. His_6_-ParB protein bound specifically to *parS2/parS3* oligonucleotide whereas at the same range of concentrations it did not form specific complexes with the control ds oligonucleotide (S1 Fig.). At ParB concentrations >5 μM, non-specific interactions with the control fragment produced smearing. To minimize non-specific binding, a competitor DNA was added in further experiments.

**Fig 3:**
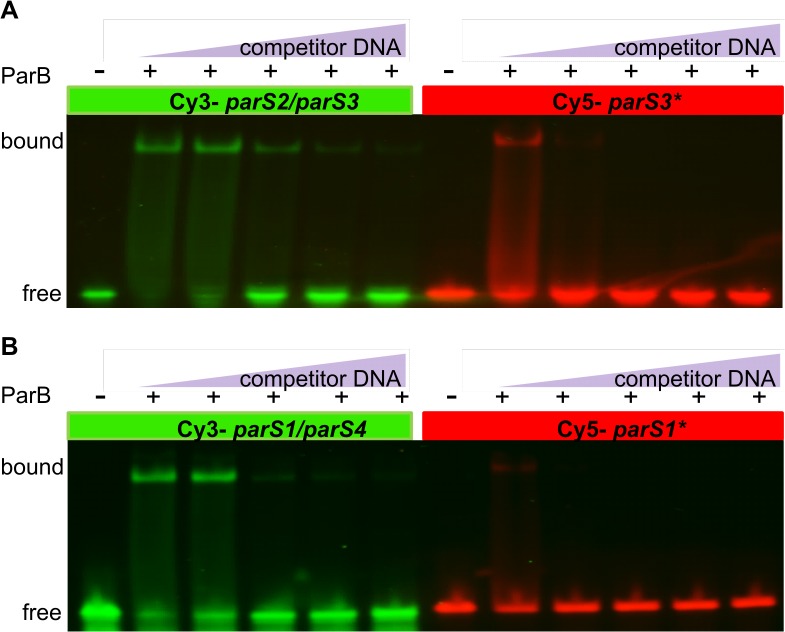
ParB binds modified parS sites with lower affinity than the wild-type counterparts. Differently labelled wt and their mutated versions were used in EMSA **(A)** *parS2/3* vs. parS3* and **(B)** *parS*1 vs. parS1*. Six pmoles of labelled nucleotides were incubated with 240 pmoles of His_6_-ParB and increasing amounts (18, 60, 90, 120, 180 pmoles) of unlabelled ds *parS2* oligonucleotide used as competitor DNA. After incubation at 37°C for 15 min, the complexes were separated on a native 5% polyacrylamide gel in TBE buffer, the DNA was visualized with FluorChemQ MultiImageIII ChemiImager and the images were captured using AlphaView software (Alpha Innotech).

**Fig 4:**
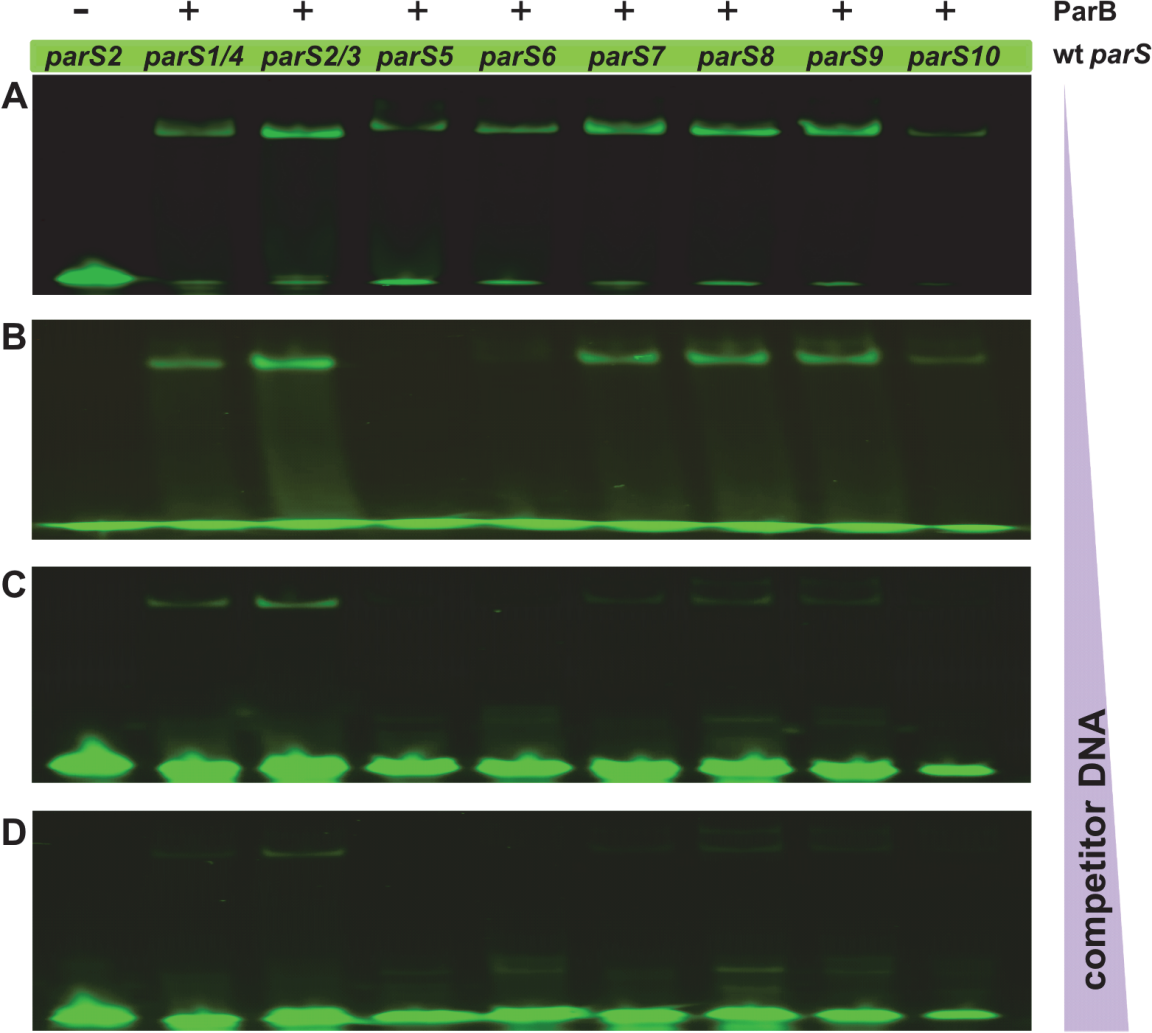
The hierarchy of ParB binding to parS sequences. Fluorescently labelled ds *parS* oligonucleotides (6 pmoles) were incubated with 240 pmoles of His_6_-ParB and increasing amounts of unlabelled ds *parS2* as competitor: **(A)** 18 pmoles **(B)** 60 pmoles **(C)** 90 pmoles and **(D)** 180 pmoles. Complexes were visualized as described in Fig. 3.

Specific ParB binding was detected for each of the wt *parS* sequences tested (data not shown). To compare affinity of ParB towards a wt *parS* and its mutated version, ParB was outcompeted from the respective complex by an excess of unlabelled wt *parS2/parS3* oligonucleotide. The results for *parS2/parS3* vs. parS3* and *parS1* vs. parS1* are shown in Fig. 3, and for *parS5—parS10* and their mutated versions in S2 Fig. This assay showed that the modified variants of almost all *parS* sequences tested were bound by ParB with an affinity 2- to 10-fold lower than were their wt counterparts. Only for the *parS5*, parS5* pair no significant difference in the ParB affinity was observed. A combination of assays for the ParB binding affinity towards different wt *parS* sequences and out-competing ParB from the complexes formed by wt *parS2/parS3* (Fig. 4) allowed us to establish the hierarchy of *in vitro* ParB binding to various wt *parS* sequences as follows: *parS2*/*parS3* > *parS1/parS4> parS*(*7–8–9*)> *parS10*> *parS6*> *parS5*.

### Construction of a library of PAO1161 *parS* mutants

**Fig 5:**
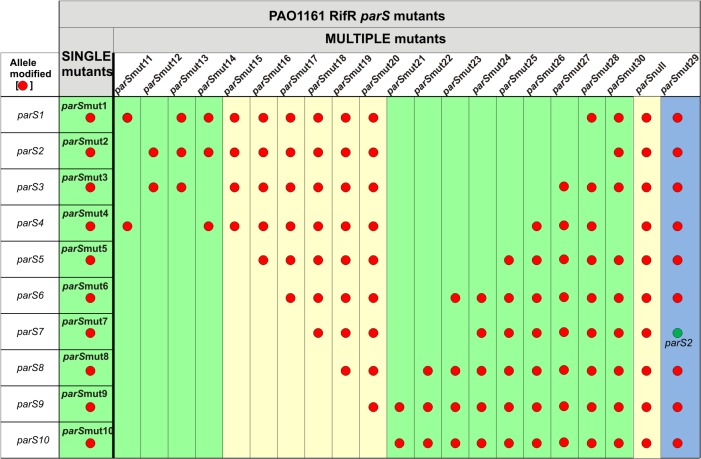
Summary of phenotypes of various *parS* mutants. The collection comprises 10 mutants with each individual *parS* modified (single) and 21 multiple mutants with between two and ten (*parS*_null_) *parS* sequences modified (red dots). Mutants in columns highlighted in green demonstrate wild-type phenotypes (wt-like), those highlighted in yellow show phenotypes similar to those observed for the *parA*_null_ and *parB*_null_ mutants (*parAB*-like). The PAO1161 Rif^R^ *parS*mut29 mutant has ectopic *parS2* (green dot) replacing parS7* in *parS*_null_ mutant (highlighted in blue).

The *in vitro* tests indicated that, with the exception of *parS5*, the introduced nucleotide substitutions (although limited in number) weakened the ParB binding. All ten pAKE600 derivatives bearing mutated *parS* sequences were introduced separately into *E*. *coli* S17–1 and the transformants were used as donors in conjugation with PAO1161 Rif^R^ [54]. Since pAKE600 derivatives are incapable of replication in *Pseudomonas*, selection for transconjugants could only pick up cells with the plasmid integrated into the chromosome by homologous recombination using the provided regions of homology (*parS* flanks). The replacement of the chromosomal wild-type *parS* sequences in the *P*. *aeruginosa* chromosome by their modified counterparts was verified by sequencing. With this approach a set of ten mutants with each single *parS* site modified was obtained (PAO1161 *parS*mut1- *parS*mut10). To construct multiple *parS* mutants we used PAO1161 *parS*mut1 or *parS*mut10 as starting strains in which all other *parS* sequences were mutated sequentially using the same allele exchange approach, until the *parS*_null_ strain with all ten sites modified was obtained (Fig. 5, S1 Table). Additionally, mutant strains PAO1161 *parS*mut12, *parS*mut13 and *parS*mut30 were constructed to address the requirement for the perfect (*parS2* and *parS3*) or nearly perfect (*parS1* and *parS4*) palindromes (Fig. 5). Finally, to study the effect of the genomic location of the perfectly palindromic *parS* sequence *parS2/parS3* was inserted into the mutated *parS7* site (opposite *oriC*) in the *parS*_null_ mutant to obtain PAO1161 *parS*mut29 (*parS*_null_ *parSmut7*::*parS2*). The thirty one PAO1161 derivatives, 10 single and 21 multiple *parS* mutants (Fig. 5, S1 Table), together with the parental strain, PAO1161 *parB*_null_ and PAO1161 *parA*_null_ mutant strains as controls were analyzed.

### Phenotypic analysis of *parS* mutants

**Fig 6:**
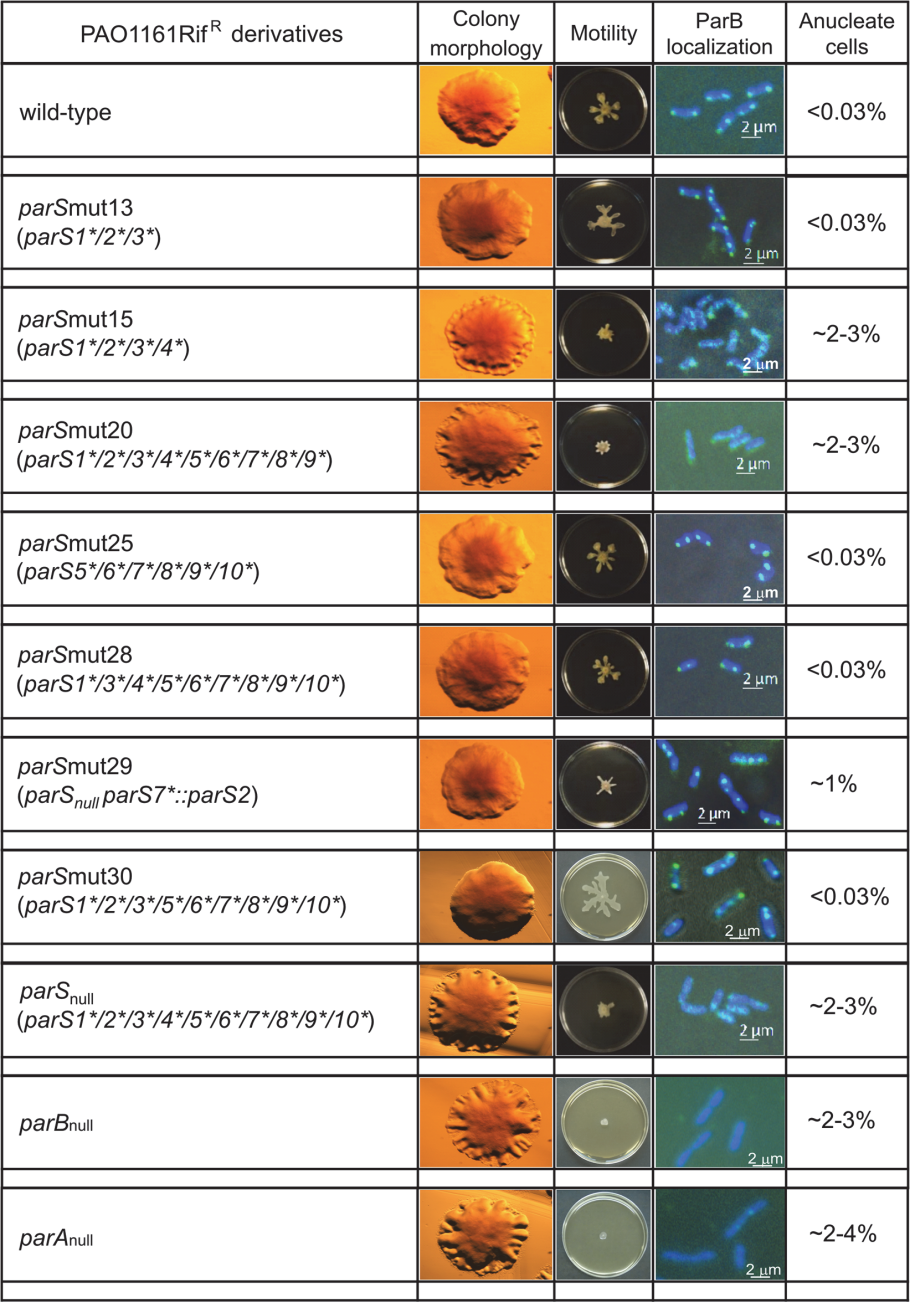
Phenotypes of selected PAO1161 *parS* mutants. Colony morphology, swarming motility, intracellular ParB localization with the use of FITC-conjugated anti-ParB antibodies, and proportion of anucleate cells after DAPI staining are shown for representative mutant strains. As the controls wt PAO1161 Rif^R^ and *parA*_null_ and *parB*_null_ mutants were tested in each set of experiments. The percentage of anucleate cells is the mean from at least three independent experiments, with approximately 1000 cells counted in each experiment.

The phenotypic characterization of the PAO1161 derivatives constructed was based on the previous analysis of the *parA* and *parB* mutants. Both *parA*_null_ and *parB*_null_ mutants of PAO1161 demonstrated a slower growth rate (10% increase in the division time), produced slightly elongated cells with up to 7% anucleate cells (during growth on rich medium), and many more cells with aberrant chromosome separation, formed wrinkled colonies and were defective in two types of motility, swarming and swimming [54, 55]. Hence, all thirty one *parS* mutants were tested for these traits and additionally, using immunofluorescence, for the ability of ParB to form foci and their subcellular localization. Since the mutants’ profiles fell into two main categories, the results are presented only for chosen *parS* mutants in Fig. 6. The first group of mutants (class wt-like, highlighted in green in Fig. 5) did not differ phenotypically from the parental strain PAO1161. The second group of *parS* mutants (class *parAB*-like, highlighted in yellow in Fig. 5) were similar in many aspects to the *parA* and *parB* mutants.

All single *parS* mutants belonged to the first group, as did multiple mutants derived from PAO1161 *parS*mut10 (*parS*mut21 to *parS*mut28), represented by PAO1161 *parS*mut25 in Fig. 6. The PAO1161 *parS*mut28 with all *parS* sites modified except *parS2* and PAO1161 *parS*mut30 with all *parS* sites modified except *parS4* had the wild-type phenotypes (Fig. 5 and Fig. 6). Notably, mutation of *parS2* in PAO1161 *parS*mut28 leading to PAO1161 *parS*_null_ strain impaired chromosome segregation, slowed the growth rate, affected motility and colony formation, and caused diffusion of the immunofluorescence signal of ParB (Figs. 5 and 6). In the second set of multiple *parS* mutants constructed in the background of PAO1161 *parS*mut1 (or *parS*mut2), neither double mutants in the sites closest to *oriC* (PAO1161 *parS*mut11 and PAO1161 *parS*mut12) nor triple mutants (PAO1161 *parS*mut13 and PAO1161 *parS*mut14) differed from the parental strain in the tests conducted (Fig. 5). The mutant phenotype appeared when all four *parS1* to *parS4* sites were modified, as in the quadruple mutant PAO1161 *parS*mut15 and all its derivatives, PAO1161 *parS*mut16 to *parS*mut20 (Figs. 5 and 6).

A comparison of two mutants with nine *parS* sequences mutated and a single *parS2* site, either present in the genome at its natural location (PAO1161 *parS*mut28) or moved to the *oriC*-distal position of *parS7* (PAO1161 *parS*mut29), revealed different phenotypes as PAO1161 *parS*mut29 strain with *parS2* at the ectopic position, only partially reversed defects of the *parS*_null_ strain. Restored swimming ability (data not shown), slightly improved swarming and wild type colony morphology, and compaction of ParB on the genome into one to four foci regularly distributed in the cell (Fig. 6), indicated the restoration of some ParB functions. However, the percentage of anucleate cells was still higher and the growth rate lower (data not shown) than for wt strain or PAO1161 *parS*mut28, indicating imperfect chromosome segregation. One should note here that introduction of *parS2/parS3* into the *parS7* site affected the coding sequence of a gene of unknown function, *pa3071*, changing two amino acid residues (E85V and R87H) in its product, but it seems unlikely that the observed defects, typical for mutants with a disturbed *par* system, resulted from those changes.

## Discussion

Chromosomal *parABS* systems participate not only in chromosome segregation but also in other fundamental processes: DNA replication, nucleoid condensation, cell division, coordination of chromosome segregation and cytokinesis, cell-to-cell communication, and motility [36]. Numerous studies have been conducted to elucidate the involvement of both Par proteins in these diverse cell functions. Here we aimed to characterize the third element of the system, the ParB-binding centromere-like *parS* sequence, in particular the roles of multiple *parS* motifs in the bacterial chromosome.

We analyzed the ParB affinity for all ten putative *parS* sequences previously identified [9] in the genome of *P*. *aerugionosa* PAO1 [69], the parental strain of the laboratory derivative PAO1161 (Fig. 1A). Eight of these sites are located within the ori domain (20% of the chromosome around *oriC*), with four clustered <16 kb from the origin of replication (*parS1-parS4*). The remaining two *parS* sequences, *parS7* and *parS8*, are located opposite *oriC* on the circular chromosome of PAO1 (∼3 Mb away).

The *in vitro* test of ParB binding to ds oligonucleotides corresponding to the wt *parS* sequences confirmed that i/ ParB binds specifically to all ten predicted *parS* sequences and ii/ there is a hierarchy of ParB binding related to the degree of divergence of the palindromic *parS* structure and the position of the mismatched nucleotides. ParB preferentially binds the perfect palindromes *parS2* and *parS3*, then with a slightly lower affinity *parS1* and *parS4* with a single mismatched pair of nucleotides. The remaining six *parS* sequences containing various double mismatches are clearly ordered. The *parS7*, *parS8* and *parS9* sequences that have one arm of the palindrome identical with the *parS2/parS3* sequence and two diverged nucleotides in the other arm are bound with a higher affinity than are *parS6* and *parS5*, in which both arms differ from *parS2/parS3* (Table 1). These data also suggest that an intact core of the palindrome (CACGTG) is important for the interactions with ParB since i/ *parS10* with two mutations in one arm within the central core demonstrates a lower binding affinity than *parS7*, *parS8* and *parS9* and ii/ *parS5* diverged in the core part of both palindromic arms is bound by ParB with the lowest affinity.

Although studies on Spo0J (ParB homologue) binding to ten *parS* sequences in the *B*. *subtilis* genome have revealed high and low affinity sites [63] as well as an asymmetry of Spo0J spreading [8], no correlation between nucleotide sequence and Spo0J affinity or direction of the spreading could be observed.

We analyzed the *P*. *aeruginosa parS* sequences in terms of 8-bp half sites and generated a sequence logo (Fig. 1C) that is slightly different from the *B*. *subtilis parS* [8] especially in the number and location of invariant positions. In *P*. *aeruginosa* only two positions are strictly conserved among the twenty *parS* half sites in contrast to four invariant nucleotides in *B*. *subtilis parS*. Additionally, the C at position 5 in the half site is invariant in *P*. *aeruginosa* but not in *B*. *subtilis* [8]. The significance of the observed species-specificity of chromosomal *parS* sequences awaits elucidation.

The ParB binding sites in the genome of PAO1161 were sequentially modified and phenotypes of the mutant strains were established to define the role of particular *parS* sequences in the biology of *P*. *aeruginosa*. Before introducing the mutated *parS* sequences into the PAO1161 chromosome effects of the nucleotide substitutions on ParB affinity were evaluated using two approaches: *in vivo*, by a test for ParB binding and spreading, so-called “transcriptional silencing” assay in a heterologous system [9], and *in vitro*, by a mobility shift assay with purified ParB. Whereas the silencing test clearly discriminated between wt and mutated versions of only *parS1*, *parS2* and *parS3*, the *in vitro* test demonstrated a decreased ParB affinity towards all mutated *parS* variants versus their wt counterparts with the exception of *parS5*. The differences in ParB binding to *parS5* and parS5* were difficult to establish due to the low ParB affinity for wt *parS5*.

For a detailed *in vivo* analysis of the role of individual *parS* sites a collection of strains with each single *parS* impaired (10 mutants) and with combinations of different mutated *parS* sequences (21 mutants) was constructed and analyzed. To the best of our knowledge, this is the first such comprehensive analysis of *parS* mutants. We assessed growth kinetics, colony morphology, motility, number of anucleate cells and the localization of ParB in the cells of the mutants. It turned out that the mutants fell into two categories, those with the wild type phenotypes (here referred to as wt-like), and those with phenotypes previously shown for *parA* and *parB* mutants [54–55] (here referred to as *parAB*-like). The defects of the latter group included slower growth (data not shown), at least a hundred-fold increased frequency of anucleate cells, dispersion of ParB foci (typical for *parA*_null_ [54] and *parB* mutants producing defective protein [55, 68]), impaired motility (swimming defects in this group of mutants were less pronounced than the defects in swarming, data not shown), and altered colony morphology. Remarkably, mutations in any single *parS* sequence did not lead to an observable defect, demonstrating functional redundancy of the *parS*s (Figs. 5 and 6). Also strains with multiple *parS* sequences modified had a wt-like phenotype as long as at least one of four *parS* from the *oriC* proximal region (*parS1-parS4*) was intact (compare *parS*mut13 and *parS*mut14 with *parS*mut15 or *parS*mut28 and *parS*mut30 with *parS*_null_, Fig. 5 and Fig. 6). However, when all four *oriC*-proximal *parS*s were mutated, a typical *parAB*-like phenotype was obtained even though the remaining six *parS* sites were unaltered; additional mutations in any or all of those sites produced no visible changes of the *parAB*-like phenotype. Since overproduction of ParB results in the same defects as a lack of ParB [9, 55] one could argue that an excess of unbound ParB was responsible for the observed defects when the four “principal” *parS* sites were impaired. However, mutation of nine out of ten *parS* sites in PAO1161 *parS*mut28 and *parS*mut30, in which only the *oriC*-proximal *parS2* and *parS4*, respectively, was left intact, still did not lead to the appearance of the *parAB*-like phenotype.

Since *parS2* and *parS3* sequences are identical as are *parS1* and *parS4* (Fig. 1B) and all four sites occupy top positions in the hierarchy of ParB binding *in vitro*, (even though *parS1/parS4* binds ParB *in vitro* with a slightly lower affinity than *parS2/parS3* as shown in Fig. 4), we conclude that i/ a single, high affinity ParB binding site from the cluster of four *parS* sequences closest to the *ori* domain (*parS1-parS4*) is necessary and sufficient for proper chromosome segregation and ii/ none of the remaining six *parS* sites can substitute for these four *oriC*-proximal sites in this function.

Since these four *parS* sequences that enable proper chromosome segregation are bound *in vitro* by ParB with the highest affinity, it was important to evaluate the role of the ParB binding strength *versus* the genomic context (localization) of the binding site. The perfect palindromic sequence *parS2* was therefore inserted in an ectopic position, 3 Mb from *oriC*, into the PAO1161 *parS*_null_ genome to give PAO1161 *parS*mut29 strain (Figs. 5 and 6). The strain with the ectopic *parS2* produced 100-fold more anucleate cells and grew slightly slower than the wt strain, indicating disturbances in chromosome segregation similar to those in the *parS*_null_ mutant. Notably, the PAO1161 *parS*mut29 strain had improved motility and colony morphology similar to wt strain. The single high affinity *parS2* site in the genome of *P*. *aeruginosa*, regardless of its genome position was also sufficient for ParB to form between one and four foci, as is typical for wt strain, confirming the model of ParB-induced condensation of chromosomal DNA by a combination of spreading and bridging after binding to the single site and undergoing a conformational change [61–62]. However, our data for *parS*mut28, *parS*mut29 and *parS*mu30 (Figs. 5 and 6) clearly indicate that the formation of a condensed ParB-DNA complex around a single *parS* will promote accurate chromosome segregation only when the complex is formed close to *oriC*, that is, on a *parS* from the *parS1-parS4* group in its native position.

The differences in functioning of *parS2* in chromosome segregation, when either at its *oriC* proximal position or distal to *oriC*, may be related to the important role of ParB-*parS* nucleoprotein complex in spatial orientation and directing the newly replicated *oriC* regions to the opposite cell poles. The *C*. *crescentus* genome has a single *parS* locus (tandem *parS* sequences) adjacent to the *parAB* operon. It has been shown that moving the *parS* region 100–400 kb away from its original position close to *oriC*, but still in the ori domain, delayed the initiation of chromosome segregation until the *parS* region had been replicated [29].

A similar experiment was conducted for *B*. *subtilis* with the ectopic *parS* inserted close to the replication terminus in a genome deprived of the eight *parS* sites from the ori domain [30], among them all six high affinity sites for Spo0J (ParB). The *B*. *subtilis* Δ8*parS* strain produced 100-fold more anucleate cells than the wt strain. The ectopic insertion of *parS* in such a mutant caused major disturbances in nucleoid organization, chromosome segregation and its coordination with cell division (a further 10-fold increase in the frequency of anucleate cells and high proportion of cells with chromosomes guillotined by the cell division septum) [30]. Since in *B*. *subtilis* one of the *parS* proximal to the *oriC* (*parS359*) is the main loading platform for the SMC condensation complex, it was hypothesized that such gross defects in the ectopic mutant strain were due to inappropriate recruitment of SMC by the Spo0J-*parS* complex formed at the replication terminus site [30]. Further studies will elucidate whether also in *P*. *aeruginosa* ParB directs SMC complex loading and if so why the defects observed in PAO1161 *parS*mut29 are not as strong as those observed for *B*. *subtilis*.

While defective in chromosome segregation and growth the PAO1161 *parS*mut29 with the single ectopic *parS2* demonstrated significantly improved motility and the wt colony morphology. This strongly suggests that various roles of ParB in the cell e.g., chromosome segregation versus regulation of gene expression may be related to its interactions with differently located *parS* sequences.

## Conclusions

The analysis of our collection of *P*. *aeruginosa parS* mutants aided by *in vitro* binding studies has revealed a hierarchy of ParB binding to different sites and a crucial role of the *parS* sequences closest to the *oriC* in accurate chromosome segregation. It has also demonstrated that a single *parS* of these four at its natural location, but not when moved opposite *oriC* in the genome, is sufficient to support accurate chromosome segregation. The role of other ParB binding sites in the biology of *P*. *aeruginosa* awaits elucidation.

## Supporting Information

S1 Fig ParB of *P*. *aeruginosa* binds to *parS* specifically. Binding reactions contained 6 pmoles of 5’ Cy3-labelled *parS2* oligonucleotide (annealed oligonucleotides #3 and #4, S3 Table) and increasing amounts of His_6_-ParB (0, 40, 80, 100 pmoles) in 20 μl of binding buffer. Cy5-labelled nonspecific oligonucleotide (annealed oligonucleotides #35 and #36) was used as a control. After incubation at 37°C for 15 min, the complexes were separated on a native 5% polyacrylamide gel in TBE buffer, the DNA was visualized with FluorChemQ MultiImageIII ChemiImager and the images were captured using AlphaView software (Alpha Innotech).
(TIF)

S2 Fig ParB binding to wt and mutated versions of *parS5-parS10*. Six pmoles of fluorescently labelled ds oligonucleotides corresponding to wt (Cy3) and mutated version (Cy5) of *parS* were incubated with 240 pmoles of His_6_-ParB and increasing amounts (18, 60, 90, 120, 180 pmoles) of the unlabelled ds wt *parS2* as competitor. **(A)** *parS5* and parS5*; **(B)** *parS6* and parS6*; **(C)** *parS7* and parS7*; **(D)** *parS8* and parS8*; **(E)** *parS9* and parS9*; **(F)** *parS10* and parS10*.
(TIF)

S1 Table Strains used in this study. (PDF)

S2 Table Plasmids used in this study. (PDF)

S3 Table Oligonucleotides used in this study. (PDF)
