# Correction: A Single *parS* Sequence from the Cluster of Four Sites Closest to *oriC* Is Necessary and Sufficient for Proper Chromosome Segregation in *Pseudomonas aeruginosa*

**DOI:** 10.1371/journal.pone.0152541

**Published:** 2016-03-24

**Authors:** Paulina Jecz, Aneta A. Bartosik, Krzysztof Glabski, Grazyna Jagura-Burdzy

In Fig 1, the table in panel B is incorrect. Please see the corrected Fig 1 here.

**Fig 1:**
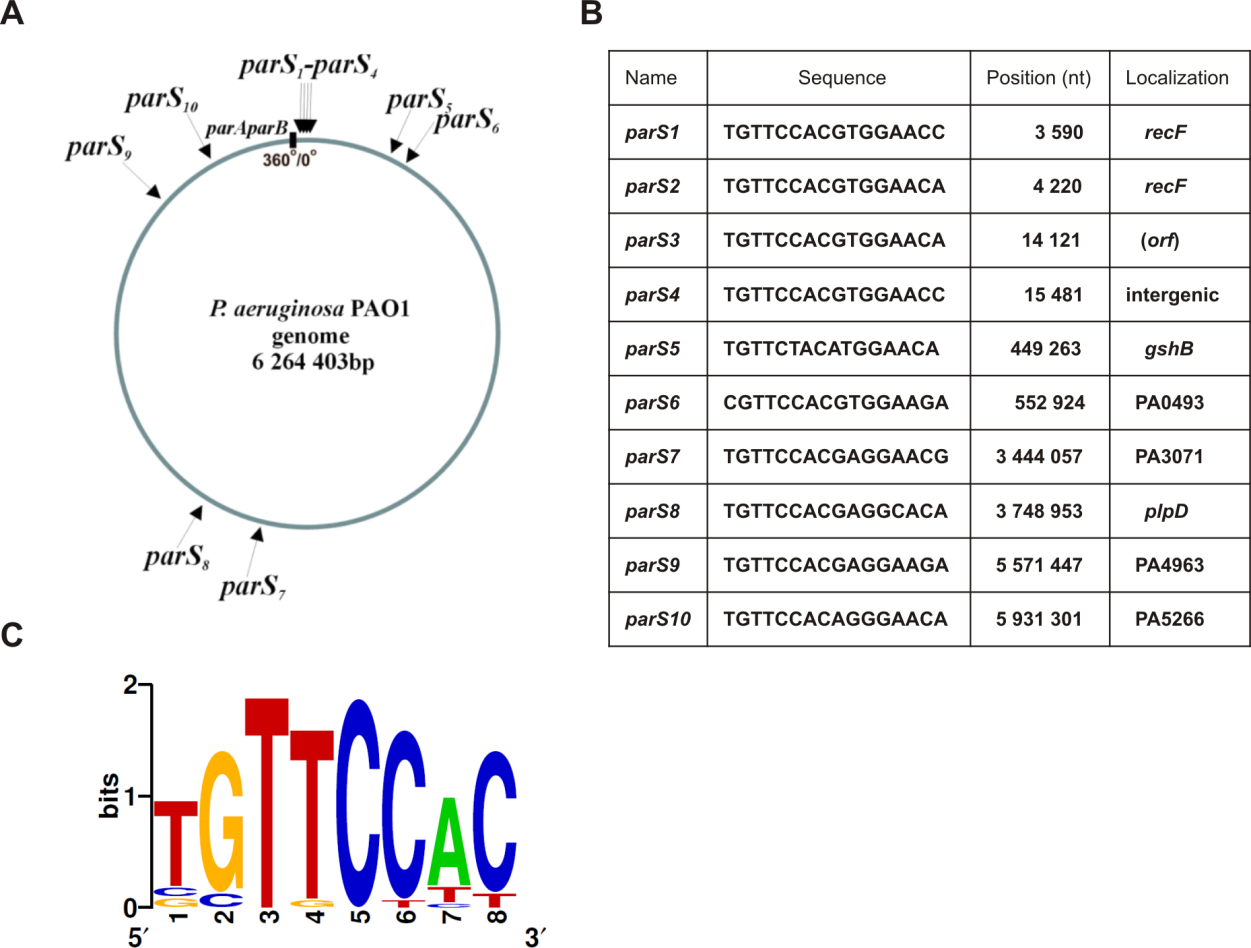
The *parS* sites and their localization in the *Pseudomonas aeruginosa* genome. **(A)** Circular map of the *P*. *aeruginosa* genome with locations of putative ParB binding sequences [9]. Position of the *parAparB* operon is shown as black rectangle, grey arrow marks *oriC*, black arrows indicate predicted *parS* sites. **(B)** Nucleotide sequences, genomic coordinates and gene locations of the *parS* sites. The sequences are presented in a clockwise configuration. The coordinates are given according to the genomic sequence of the PAO1-UW strain [69]. **(C)** Sequence logo for all twenty 8-bp half-sites in the *P*. *aeruginosa* PAO1-UW genome (weblogo.berkeley.edu/logo.cgi). Nucleotides at positions 2 and 5 are invariant in all half-sites.
