# Supplementary figures and images for: A Single parS Sequence from the Cluster of Four Sites Closest to oriC Is Necessary and Sufficient for Proper Chromosome Segregation in Pseudomonas aeruginosa
Source: PLoS One. 2015 Mar 20;10(3):e0120867. doi: 10.1371/journal.pone.0120867 (PMC4368675; doi:10.1371/journal.pone.0120867)

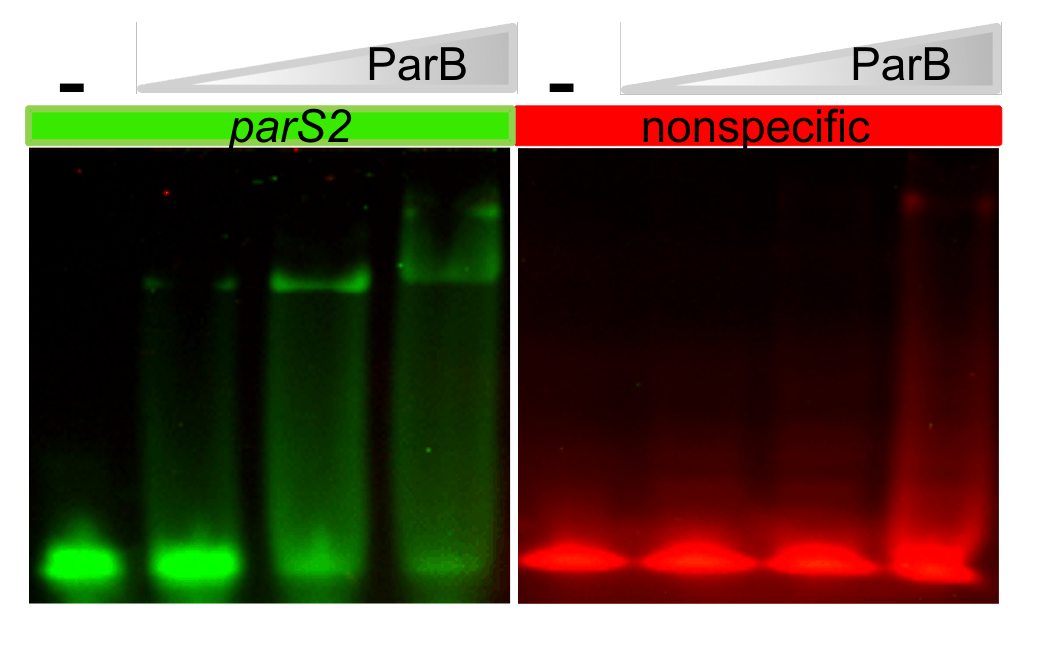

Supplement: S1 Fig — Binding reactions contained 6 pmoles of 5’ Cy3-labelled parS2 oligonucleotide (annealed oligonucleotides #3 and #4, S3 Table) and increasing amounts of His6-ParB (0, 40, 80, 100 pmoles) in 20 μl of binding buffer. Cy5-labelled nonspecific oligonucleotide (annealed oligonucleotides #35 and #36) was used as a control. After incubation at 37°C for 15 min, the complexes were separated on a native 5% polyacrylamide gel in TBE buffer, the DNA was visualized with FluorChemQ MultiImageIII ChemiImager and the images were captured using AlphaView software (Alpha Innotech). (TIF) [file pone.0120867.s001.tif]

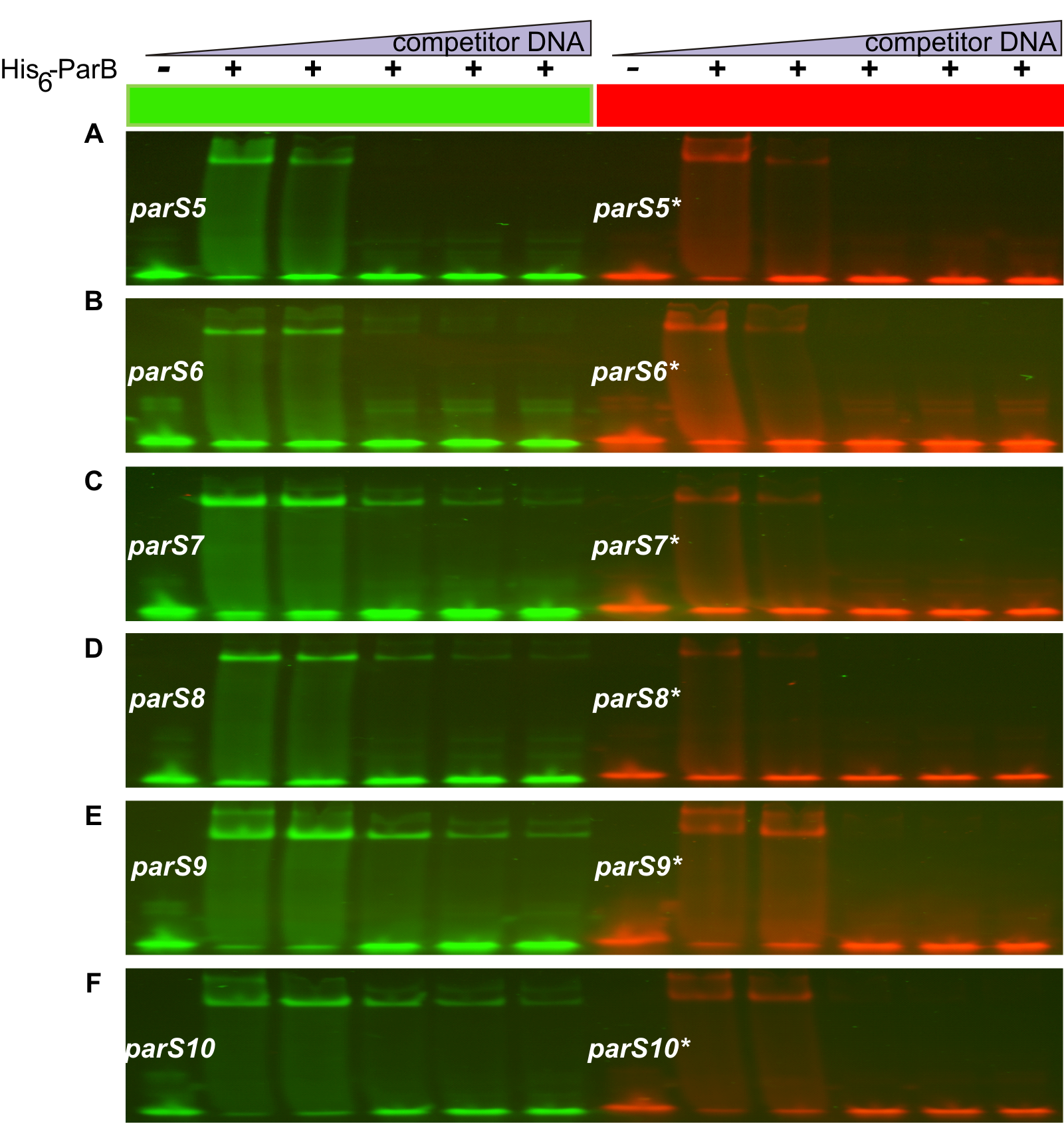

Supplement: S2 Fig — Six pmoles of fluorescently labelled ds oligonucleotides corresponding to wt (Cy3) and mutated version (Cy5) of parS were incubated with 240 pmoles of His6-ParB and increasing amounts (18, 60, 90, 120, 180 pmoles) of the unlabelled ds wt parS2 as competitor. (A) parS5 and parS5*; (B) parS6 and parS6*; (C) parS7 and parS7*; (D) parS8 and parS8*; (E) parS9 and parS9*; (F) parS10 and parS10*. (TIF) [file pone.0120867.s002.tif]
