# Supplementary material for: A Single parS Sequence from the Cluster of Four Sites Closest to oriC Is Necessary and Sufficient for Proper Chromosome Segregation in Pseudomonas aeruginosa
Source: PLoS One. 2015 Mar 20;10(3):e0120867. doi: 10.1371/journal.pone.0120867 (PMC4368675; doi:10.1371/journal.pone.0120867)
Supplement: S1 Table — (PDF) [file pone.0120867.s003.pdf]

S1_Table. Strains used in this study.

Strain	Genotype	Source
Escherichia coli		
BL21 (DE3)	F^- *ompT hsdS*$_B$(r_B^- m_B^-) *gal dcm* (λDE3)	Novagen Inc.
DH5α	*recA1 endA1 gyrA96 thi-1 hsdR17*(r_K^- m_K^+) *supE44 relA1 deoR* Δ(*lacZYA-argF*)*U169* (φ80d*lacZ* ΔM15)	[1] [a]
S17-1	*pro* Δ*hsdR hsdM*$^+$ *recA* Tp^R Sm^R ΩRP4-Tc::Mu-Kn::Tn7	[2] [a]
Pseudomonas aeruginosa		
PAO1161 Rif^R	*leu* r^- Rif^R	[3] [a]
PAO1161 Rif^R *parB*$_{null}$	*leu* r^- Rif^R *parB1-18::Tc*^{R}	[4] [a]
PAO1161 Rif^R *parA*$_{null}$	*leu* r^- Rif^R *parA1-39::Sm*^{R}	[3] [a]
PAO1161 Rif^R *parS*mut1	*parS1**, allele exchange in PAO1161 Rif^R with the use of pPJB11	This study
PAO1161 Rif^R *parS*mut2	*parS2**, allele exchange in PAO1161 Rif^R with the use of pPJB12	This study
PAO1161 Rif^R *parS*mut3	*parS3**, allele exchange in PAO1161 Rif^R with the use of pPJB13	This study
PAO1161 Rif^R *parS*mut4	*ΔparS4**, allele exchange in PAO1161 Rif^R with the use of pPJB14	This study
PAO1161 Rif^R *parS*mut5	*parS5**, allele exchange in PAO1161 Rif^R with the use of pPJB15	This study
PAO1161 Rif^R *parS*mut6	*parS6**, allele exchange in PAO1161 Rif^R with the use of pPJB16	This study
PAO1161 Rif^R *parS*mut7	*parS7**, allele exchange in PAO1161 Rif^R with the use of pPJB17	This study
PAO1161 Rif^R *parS*mut8	*parS8**, allele exchange in PAO1161 Rif^R with the use of pPJB18	This study
PAO1161 Rif^R *parS*mut9	*parS9**, allele exchange in PAO1161 Rif^R with the use of pPJB19	This study
PAO1161 Rif^R *parS*mut10	*parS10**, allele exchange in PAO1161 Rif^R with the use of pPJB20	This study
PAO1161 Rif^R *parS*mut11	*parS1**, Δ*parS4**, allele exchange in PAO1161 Rif^R *parS*mut1 with the use of pPJB14	This study
PAO1161 Rif^R *parS*mut12	*parS2**, *parS3**, allele exchange in PAO1161 Rif^R *parS*mut2 with the use of pPJB13	This study
PAO1161 Rif^R *parS*mut13	*parS1**, *parS2**, *parS3**, allele exchange in PAO1161 Rif^R *parS*mut12 with the use of pPJB11	This study
PAO1161 Rif^R *parS*mut14	*parS1* and parS2**, Δ*parS4*, allele exchange in PAO1161 Rif^R *parS*mut11 with the use of pPJB12	This study
PAO1161 Rif^R *parS*mut15	*parS1**, *parS2**, *parS3**, Δ*parS4*, allele exchange in PAO1161 Rif^R *parS*mut14 with the use of pPJB13	This study
PAO1161 Rif^R *parS*mut16	*parS1**, *parS2**, *parS3** Δ*parS4*, *parS5**, allele exchange in PAO1161 Rif^R *parS*mut15 with the use of pPJB15	This study

PAO1161 Rif^R *parS*mut17	*parS1**, *parS2**, *parS3**, Δ*parS4*, *parS5**, *parS6**, allele exchange in PAO1161 Rif^R *parS*mut16 with the use of pPJB16	This study
PAO1161 Rif^R *parS*mut18	*parS1**, *parS2**, *parS3**, Δ*parS4*, *parS5**, *parS6**, *parS7*, allele exchange in PAO1161 Rif^R *parS*mut17 with the use of pPJB17	This study
PAO1161 Rif^R *parS*mut19	*parS1**, *parS2**, *parS3**, Δ*parS4*, *parS5**, *parS6**, *parS7**, *parS8**, allele exchange in PAO1161 Rif^R *parS*mut18 with the use of pPJB18	This study
PAO1161 Rif^R *parS*mut20	*parS1**, *parS2**, *parS3**, Δ*parS4*, *parS5**, *parS6**, *parS7**, *parS8**, *parS9**, allele exchange in PAO1161 Rif^R *parS*mut19 with the use of pPJB19	This study
PAO1161 Rif^R *parS*mut21	*parS9**, *parS10**, allele exchange in PAO1161 Rif^R *parS*mut10 with the use of pPJB19	This study
PAO1161 Rif^R *parS*mut22	*parS8**, *parS9**, *parS10**,allele exchange in PAO1161 Rif^R *parS*mut22 with the use of pPJB18	This study
PAO1161 Rif^R *parS*mut23	*parS6**, *parS8**, *parS9**, *parS10**, allele exchange in PAO1161 Rif^R *parS*mut23 with the use of pPJB16	This study
PAO1161 Rif^R *parS*mut24	*parS6**, *parS7**, *parS8**, *parS9**, *parS10**, allele exchange in PAO1161 Rif^R *parS*mut24 with the use of pPJB17	This study
PAO1161 Rif^R *parS*mut25	*parS5** *parS6**, *parS7**, *parS8**, *parS9**, *parS10**, allele exchange in PAO1161 Rif^R *parS*mut25 with the use of pPJB15	This study
PAO1161 Rif^R *parS*mut26	*ΔparS4**, *parS5** *parS6**, *parS7**, *parS8**, *parS9**, *parS10**, allele exchange in PAO1161 Rif^R *parS*mut25 with the use of pPJB14	This study
PAO1161 Rif^R *parS*mut27	*parS1**, *ΔparS4**, *parS5** *parS6**, *parS7**, *parS8**, *parS9**, *parS10**, allele exchange in PAO1161 Rif^R *parS*mut26 with the use of pPJB11	This study
PAO1161 Rif^R *parS*mut28	*parS1**, *parS3**, Δ*parS4*, *parS5**, *parS6**, *parS7**, *parS8**, *parS9**, *parS10**, allele exchange in PAO1161 Rif^R *parS*mut27 with the use of pPJB13	This study
PAO1161 Rif^R *parS*mut29	*parS7** replaced with *parS2*, allele exchange in PAO1161 Rif^R *parS*$_{null}$ with the use of pPJB21	This study
PAO1161 Rif^R *parS*mut30	*parS1**, *parS2**, *parS3**, *parS5**, *parS6**, *parS7**, *parS8**, *parS9**, *parS10**, allele exchange in PAO1161 Rif^R *parS*$_{null}$ with the use of pPJB22	This study
PAO1161 Rif^R *parS*$_{null}$	*parS1**, *parS2**, *parS3**, Δ*parS4*, *parS5**, *parS6**, *parS7**, *parS8**, *parS9**, *parS10**, allele exchange in PAO1161 Rif^R *parS*mut20 with the use of pPJB20	This study

[a] References:

1. Hanahan D. Studies on transformation of *Escherichia coli* with plasmids. J Mol Biol. 1983;166: 557 – 580. doi:http://dx.doi.org/10.1016/S0022-2836(83)80284-8

2. Simon R, Priefer U, Puhler A. A broad host range mobilization system for *in vivo* genetic engineering: transposon mutagenesis in Gram negative bacteria. Nat Biotechnol. 1983;1: 784–791. doi:10.1038/nbt1183-784

3. Lasocki K, Bartosik AA, Mierzejewska J, Thomas CM, Jagura-Burdzy G. Deletion of the *parA* (*soj*) homologue in *Pseudomonas aeruginosa* causes ParB instability and affects growth rate, chromosome segregation, and motility. J Bacteriol. 2007;189: 5762–5772. doi:10.1128/JB.00371-07

4. Bartosik AA, Mierzejewska J, Thomas CM, Jagura-Burdzy G. ParB deficiency in *Pseudomonas aeruginosa* destabilizes the partner protein ParA and affects a variety of physiological parameters. Microbiology. 2009;155: 1080–1092. doi:10.1099/mic.0.024661-0
