# Supplementary material for: A Single parS Sequence from the Cluster of Four Sites Closest to oriC Is Necessary and Sufficient for Proper Chromosome Segregation in Pseudomonas aeruginosa
Source: PLoS One. 2015 Mar 20;10(3):e0120867. doi: 10.1371/journal.pone.0120867 (PMC4368675; doi:10.1371/journal.pone.0120867)
Supplement: S2 Table — (PDF) [file pone.0120867.s004.pdf]

S2_Table. Plasmids used in this study.

Plasmid	Relevant features	Source
Plasmids provided by others		
pABB812	HindIII-SalI fragment carrying *parS2/3* sequence (orientation II) inserted into pGB2	[1] [a]
pABB822	HindIII-SalI fragment carrying *parS1/4* sequence (orientation II) inserted into pGB2	[1] [a]
pABB832	HindIII-SalI fragment carrying *parS7* sequence (orientation II) inserted into pGB2	[1] [a]
pAKE600	ori_{MB1} $ori\mathrm{T}_{\mathrm{RK2}}$; Ap^{R} *sacB*	[2] [a]
pET28mod	ori_{MB1} Km^{R}, T7*p*, *lacO*, His tag, no BamHI site	[3] [a]
pGB2	ori_{SC101}; $\mathrm{Sp}^{\mathrm{R}}/\mathrm{Sm}^{\mathrm{R}}$	[4] [a]
pGBT30	ori_{MB1} Ap^{R}, $lacI^{q}$, *tacp* expression vector	[5] [a]
pKLB2	ori_{MB1}, Ap^{R}, $lacI^{q}$, *tacp-*$parB_{\mathrm{P.a.}}$	[1] [a]
pKLB28	pET28mod T7*p*- $parB_{\mathrm{P.a.}}$	[1] [a]
Plasmids constructed		
pAKE600 derivatives		
pPJB1	*parS1*, 500 nt EcoRI-SalI fragment amplified with #57 and #58 primers on the PAO1161 genomic DNA as a template	This study
pPJB2	*parS2*, 500 nt EcoRI-SalI fragment amplified with #59 and #60 primers on the PAO1161 genomic DNA as a template	This study
pPJB3	*parS3*, 500 nt EcoRI-SalI fragment amplified with #61 and #62 primers on the PAO1161 genomic DNA as a template	This study
pPJB4.1	250 nt fragment upstream from *parS4*, inserted as EcoRI-NcoI after PCR amplification with #63 and #64 primers on the PAO1161 genomic DNA as a template	This study
pPJB4.2	250 nt fragment downstream from *parS4*, inserted as NcoI-XhoI after PCR amplification with #65 and #66 primers on the PAO1161 genomic DNA as a template	This study
pPJB5	*parS5*, 500nt XbaI-SmaI fragment amplified with #67 and #68 primers on the PAO1161 genomic DNA as a template	This study
pPJB6	*parS6*, 500nt EcoRI-SalI fragment amplified with #69 and #70 primers on the PAO1161 genomic DNA as a template	This study
pPJB7	*parS7*, 500nt EcoRI-NcoI fragment amplified with #71 and #72 primers on the PAO1161 genomic DNA as a template	This study
pPJB8	*parS8*, 500nt EcoRI-NcoI fragment amplified with #73 and #74 primers on the PAO1161 genomic DNA as a template	This study
pPJB9	*parS9*, 500nt EcoRI-SalI fragment amplified with #75 and #76 primers on the PAO1161 genomic DNA as a template	This study
pPJB10	*parS10*, 500nt EcoRI-NcoI fragment amplified with #77 and #78 primers on the PAO1161 genomic DNA as a template	This study
pPJB11	*parS1**[b], PCR site-directed mutagenesis of pPJB1 with #79 and #80 primers	This study
pPJB12	*parS2**, PCR site-directed mutagenesis of pPJB2 with #81 and #82 primers	This study
pPJB13	*parS3**, PCR site-directed mutagenesis of pPJB3 with #83 and #84 primers	This study
pPJB14	*ΔparS4*, NcoI-XhoI fragment of pJB4.2 inserted into pJB4.1	This study

pPJB15	*parS5**, PCR site-directed mutagenesis of pPJB5 with #85 and #86 primers	This study
pPJB16	*parS6**, PCR site-directed mutagenesis of pPJB6 with #87 and #88 primers	This study
pPJB17	*parS7**, PCR site-directed mutagenesis of pPJB7 with #89 and #90 primers	This study
pPJB18	*parS8**, PCR site-directed mutagenesis of pPJB8 with #91 and #92 primers	This study
pPJB19	*parS9**, PCR site-directed mutagenesis of pPJB9 with #93 and #94 primers	This study
pPJB20	*parS10**, PCR site-directed mutagenesis of pPJB10 with #95 and #96 primers	This study
pPJB21	*parS7* modified into *parS2*, PCR site-directed mutagenesis of pPJB7 with #97 and #98 primers	This study
pPJB22	*parS4*, 500 nt EcoRI-XhoI fragment amplified with #63 and #66 primers on the PAO1161 genomic DNA as a template and inserted between EcoRI and SalI sites	This study
pGB2 derivatives		
pPJB22	*parS5*, annealed oligonucleotides #41 and #6 inserted between EcoRI-SalI sites	This study
pPJB23	*parS6*, annealed #42 and #8 oligonucleotides inserted between EcoRI-SalI sites	This study
pPJB24	*parS8*, annealed #45 and #12 oligonucleotides inserted between EcoRI-SalI sites	This study
pPJB25	*parS9*, annealed #46 and #14 oligonucleotides inserted between EcoRI-SalI sites	This study
pPJB26	*parS10*, annealed #47 and #16 oligonucleotides inserted between EcoRI-SalI sites	This study
pPJB27	*parS1**, annealed #48 and #18 oligonucleotides inserted between EcoRI-SalI sites	This study
pPJB28	*parS2**, annealed #49 and #20 oligonucleotides inserted between EcoRI-SalI sites	This study
pPJB29	*parS3**, annealed #50 and #22 oligonucleotides inserted between EcoRI-SalI sites	This study
pPJB30	*parS5**, annealed #51 and #24 oligonucleotides inserted between EcoRI-SalI sites	This study
pPJB31	*parS6**, annealed #52 and #26 oligonucleotides inserted between EcoRI-SalI sites	This study
pPJB32	*parS7**, annealed #53 and #28 oligonucleotides inserted between EcoRI-SalI sites	This study
pPJB33	*parS8**, annealed #54 and #30 oligonucleotides inserted between EcoRI-SalI sites	This study
pPJB34	*parS9**, annealed #55 and #32 oligonucleotides inserted between EcoRI-SalI sites	This study
pPJB35	*parS10**, annealed #56 and #34 oligonucleotides inserted between EcoRI-SalI sites	This study

[a] References

1. Bartosik AA, Lasocki K, Mierzejewska J, Thomas CM, Jagura-Burdzy G. ParB of *Pseudomonas aeruginosa*: interactions with its partner ParA and its target *parS* and specific effects on bacterial growth. J Bacteriol. 2004;186: 6983–6998. doi:10.1128/JB.186.20.6983-6998.2004

2. El-Sayed AK, Hothersall J, Thomas CM. Quorum-sensing-dependent regulation of biosynthesis of the polyketide antibiotic mupirocin in *Pseudomonas fluorescens* NCIMB 10586. Microbiology. 2001;147: 2127–2139.

3. Lukaszewicz M, Kostelidou K, Bartosik AA, Cooke GD, Thomas CM, Jagura-Burdzy G. Functional dissection of the ParB homologue (KorB) from IncP-1 plasmid RK2. Nucleic Acids Res. 2002;30: 1046–1055. doi:10.1093/nar/30.4.1046

4. Churchward G, Belin D, Nagamine Y. A pSC101-derived plasmid which shows no sequence homology to other commonly used cloning vectors. Gene. 1984;31: 165 – 171. doi:http://dx.doi.org/10.1016/0378-1119(84)90207-5

5. Jagura-Burdzy G, Ibbotson JP, Thomas CM. The *korF* region of broad-host-range plasmid RK2 encodes two polypeptides with transcriptional repressor activity. J Bacteriol. 1991;173: 826–833.

[b]- * corresponds to the mutated version of *parS* sequence as indicated in Table 1
