# Supplementary material for: A Single parS Sequence from the Cluster of Four Sites Closest to oriC Is Necessary and Sufficient for Proper Chromosome Segregation in Pseudomonas aeruginosa
Source: PLoS One. 2015 Mar 20;10(3):e0120867. doi: 10.1371/journal.pone.0120867 (PMC4368675; doi:10.1371/journal.pone.0120867)
Supplement: S3 Table — (PDF) [file pone.0120867.s005.pdf]

S3_Table. Oligonucleotides used in this study.

No	Oligonucleotide	Sequence
		**EMSA**
#1	parS1/4Fa	Cy3-AATTCAAGCTT**TGTTCCACGTGGAACC**G
#2	parS1/4Ra	TCGAC**GGTTCCACGTGGAACA**AAGCTTG
#3	parS2/3Fa	Cy3- AATTCAAGCTT**TGTTCCACGTGGAACA**G
#4	parS2/3Ra	TCGAC**TGTTCCACGTGGAACA**AAGCTTG
#5	parS5Fa	Cy3-AATTCAAGCTT**TGTTCTACATGGAACA**G
#6	parS5Ra	TCGAC**TGTTCCATGTAGAACA**AAGCTTG
#7	parS6Fa	Cy3-AATTCAAGCTT**CGTTCCACGTGGAAGA**G
#8	parS6R	TCGAC**TCTTCCACGTGGAACG**AAGCTTG
#9	parS7Fa	Cy3-AATTCAAGCTT**TGTTCCACGAGGAACC**G
#10	parS7Ra	TCGAC**CGTTCCTCGTGGAACA**AAGCTTG
#11	parS8Fa	Cy3-AATTCAAGCTT**TGTTCCACGAGGCACA**G
#12	parS8R	TCGAC**TGTGCCTCGTGGAACA**AAGCTTG
#13	parS9Fa	Cy3-AATTCAAGCTT**TGTTCCACGAGGAAGA**G
#14	parS9R	TCGAC**TCTTCCTCGTGGAACA**AAGCTTG
#15	parS10Fa	Cy3-AATTCAAGCTT**TGTTCCACAGGGAACA**G
#16	parS10R	TCGAC**TGTTCCCTGTGGAACA**AAGCTTG
#17	pS1mutFa	Cy5-AATTCAAGCTT**TATTTCATGTAGAGCC**G
#18	pS1mutR	TCGAC**GGCTCTACATGAAATA**AAGCTTG
#19	pS2mutFa	Cy5-AATTCAAGCTT**TGTTTCATGTAGAGCA**G
#20	pS2mutR	TCGAC**TGCTCTACATGAAACA**AAGCTTG
#21	pS3mutFa	Cy5-AATTCAAGCTT**CGTGCCCCGAGGGACG**G
#22	pS3mutR	TCGAC**CGTCCCTCGGGGCACG**AAGCTTG
#23	pS5mutFa	Cy5-AATTCAAGCTT**TATTGTATATGGAGCA**G
#24	pS5mutR	TCGAC**TGCTCCATATACAATA**AAGCTTG
#25	pS6mutFa	Cy5-AATTCAAGCTT**CGTCCCTCGCGGCAGG**G
#26	pS6mutR	TCGAC**CCTGCCGCGAGGGACG**AAGCTTG
#27	pS7mutFa	Cy5-AATTCAAGCTT**TATTTCATGAAGAGCG**G
#28	pS7mutR	TCGAC**CGCTCTTCATGAAATA**AAGCTTG
#29	pS8mutFa	Cy5-AATTCAAGCTT**TATTTCATGAAGCGCA**G
#30	pS8mutR	TCGAC**TGCGCTTCATGAAATA**AAGCTTG
#31	pS9mutFa	Cy5-AATTCAAGCTT**TATTTCATGAAGAGGA**G
#32	pS9mutR	TCGAC**TCCTCTTCATGAAATA**AAGCTTG
#33	pS10mutFa	Cy5-AATTCAAGCTT**TATTTCATAGAGAGCA**G
#34	pS10mutR	TCGAC**TGCTCTCTATGAAATA**AAGCTTG
#35	nonspecificF	Cy5-GATAGCTCTTTGCCATTAAC
#36	nonspecificR	GTTAATGGCAAAGAGCTATC
		**SILENCING**
#37	parS1/4Fb	AGCTTGTTGCT**TGTTCCACGTGGAACC**AGGCCG
#38	parS1/4Rb	TCGACGGCCT**GGTTCCACGTGGAACA**GCAACA
#39	parS2/3Fb	AGCTTGTTGCT**TGTTCCACGTGGAACA**AGGCCG
#40	parS2/3Rb	TCGACGGCCT**TGTTCCACGTGGAACA**GCAACA
#41	parS5Fb	AATTCAAGCTT**TGTTCTACATGGAACA**G

#42	parS6Fb	AATTCAAGCTT**CGTTCCACGTGGAAGA**G
#43	parS7Fb	AGCTTGTTGCT**TGTTCCACGAGGAACG**AGGCCG
#44	parS7Rb	TCGACGGCCT**CGTTCCTCGTGGAACA**GCAACA
#45	parS8Fb	AATTCAAGCTT**TGTTCCACGAGGCACA**G
#46	parS9Fb	AATTCAAGCTT**TGTTCCACGAGGAAGA**G
#47	parS10Fb	AATTCAAGCTT**TGTTCCACAGGGAACA**G
#48	pS1mutFb	AATTCAAGCTT**TATTTCATGTAGAGCC**G
#49	pS2mutFb	AATTCAAGCTT**TGTTTCATGTAGAGCA**G
#50	pS3mutFb	AATTCAAGCTT**CGTGCCCCGAGGGACG**G
#51	pS5mutFb	AATTCAAGCTT**TATTGTATATGGAGCA**G
#52	pS6mutFb	AATTCAAGCTT**CGTCCCTCGCGGCAGG**G
#53	pS7mutFb	AATTCAAGCTT**TATTTCATGAAGAGCG**G
#54	pS8mutFb	AATTCAAGCTT**TATTTCATGAAGCGCA**G
#55	pS9mutFb	AATTCAAGCTT**TATTTCATGAAGAGGA**G
#56	pS10mutFb	AATTCAAGCTT**TATTTCATAGAGAGCA**G
**PCR**		
#57	parS1F	GCGAATTCAAGCGGCCTGCACCGTATTC
#58	parS1R	CGGTCGACCTTGTCCCAGCCTCGGTAGT
#59	parS2F	GCGAATTCGCAGCGTGCGGATCTTCGCA
#60	parS2R	CGGTCGACGGCGTGGAGGACGGTCATGA
#61	parS3F	GCGAATTCCGACGAACTGATGCTCGACC
#62	parS3R	CGGTCGACGGGGAAGCACTTGGACAGGT
#63	parS4AR	GCGAATTCCCGACAATACCCACGAACCCC
#64	parS4AF	CGCCATGGTCACCGTTCAGGCTTCGCTG
#65	parS4BF	GCCCATGGCCAGGCATGAGCCACTGATC
#66	parS4BR	CGCTCGAGGAAACCACGGAACGCCGCTT
#67	parS5F	GCTCTAGATAACGCCCTGTCACCGACGG
#68	parS5R	CGCCCGGGTATCGGATCGCCGGCTGACC
#69	parS6F	GCGAATTCGCGAGCCTCGCGACAAGCTT
#70	parS6R	CGGTCGACTCCCGCGCCGCCATGAGAAT
#71	parS7F	GCGAATTCGGCCTCGACGAGCTTCTGGA
#72	parS7R	CGCCATGGCGCGATTGGCCCGCAATAGC
#73	parS8F	GCGAATTCTGCAGGACGACATGAGCACCCTCTA
#74	parS8R	CGCCATGGTCGTGGGGGAAATTGACGTCGTCGA
#75	parS9F	GCGAATTCGGAGACCAATGTCAGCCAGC
#76	parS9R	CGGTCGACTGCCACTGGTGGTGCCGATTC
#77	parS10F	GCGAATTCCCTGGGAGGCGTTCTTCCGT
#78	parS10R	CGCCATGGACGAACTGGCCGCTGGCGAT
**PCR-based site directed mutagenesis**		
#79	pS1mutF	TGGGGAG**TATTTCATGTAGAGCC**TCGGTTTCTGCCC
#80	pS1mutR	CAGAAA**CCGAGGCTCTACATGAAATA**CTCCCCAATC
#81	pS2mutF	CGCCGGTATCCA**TGTTTCATGTAGAGCA**TGGAAAAGTCTC
#82	pS2mutR	GAGACTTTTCCA**TGCTCTACATGAAACA**TGGATACCGGCG
#83	pS3mutF	CGTTCGGA**CGTGCCCCGAGGGACG**TCGGCGCGCAGCATA
#84	pS3mutR	TATGCTGCGCGCCGA**CGTCCCTCGGGGCACG**TCCGAACG

#85	pS5mutF	CCAGGCCCGGGGCTGGTCGC**TATTGTATATGGAGCA**GCAGGACCT
#86	pS5mutR	TCCTGC**TGCTCCATATAAAATA**GCGACCAGCCCCGGCCTGGGCA
#87	pS6mutF	CATACTCCAGG**CGTCCCTCGCGGCAGG**TGGCGAGGCC
#88	pS6mutR	GGCCTCGCCA**CCTGCCGCGAGGGACG**CCTGGAGTATG
#89	pS7mutF	GCACACCAAGC**TATTTCATGAAGAGCG**CGAGCGTCCGGTGTTCGTCC
#90	pS7mutR	GGACGAACACCGGACGCTCG**CGCTCTTCATGAAATA**GCTTGGTGTGC
#91	pS8mutF	CGGCTCGCGATACTTCGTCGCGCCCTTCC**TATTTCATGAAGCGCA**GAACGTC
#92	pS8mutR	GTTC**TGCGCTTCATGAAATA**GGAAGGGCGCGACGAAGTATCGCGAGCCGACG
#93	pS9mutF	AGAAGGGAGATCTGAAGGACAGCCGGG**TATTTCATGAAGAGGA**ACAGCATC
#94	pS9mutR	GCTGT**TCCTCTTCATGAAATA**CCCGGCTGTCCTTCAGATCTCCCTTCTGGT
#95	pS10mutF	CCGCTGGTCACCGGTTGCC**TATTTCATAGAGAGCA**TTCCGGTGCCC
#96	pS10mutR	CACCGGA**TGCTCTCTATGAAATA**GGCAACCGGTGACCAGCGGCTG
#97	pS7-2modF	CACCAAGC**TGTTCCACGTGGAACA**CGAGCGACCGGTG
#98	pS7-2modR	CACCGGTCGCTCG**TGTTCCACGTGGAACA**GCTTGGTG

Sequences corresponding to the *parS/parS** are in bold, the restriction sites destroyed or introduced during PCR mutagenesis are underlined.
